# The effect of mental countermeasures on a novel brain‐based feedback concealed information test

**DOI:** 10.1002/hbm.25814

**Published:** 2022-02-23

**Authors:** Jinbin Zheng, Jiayu Cheng, Chongxiang Wang, Xiaohong Lin, Genyue Fu, Liyang Sai

**Affiliations:** ^1^ Department of Psychology Hangzhou Normal University Hangzhou China; ^2^ Center for Cognition and Brain Disorders The Affiliated Hospital of Hangzhou Normal University Hangzhou China; ^3^ Zhejiang Key Laboratory for Research in Assessment of Cognitive Impairments Hangzhou China

**Keywords:** concealed information, deception, feedback, lie detection, P300

## Abstract

The feedback concealed information test (fCIT) is a novel form of the CIT, providing participants with feedback regarding their memory concealment performance. The fCIT utilizes event‐related potentials (recognition‐P300 and feedback‐related event‐related potentials) and has been shown to provide high efficiency in detecting information concealment. However, it is unclear how well the fCIT performs in the presence of mental countermeasures. To address this question, participants were trained to use countermeasures during fCIT. Results showed that the recognition‐P300 efficiency decreased when participants used countermeasures. However, the efficiencies of feedback‐related negativity and feedback‐P300 were unchanged, with feedback‐P300 still showing a high detection efficiency (AUC = 0.86) during countermeasures. These findings demonstrate the potential of fCIT for subverting countermeasures.

## Graphical abstract

The present results showed that the recognition‐P300 efficiency decreased when participants used countermeasures. However, the efficiencies of feedback‐related negativity (FRN) and feedback‐P300 were unchanged, with feedback‐P300 still showing a high detection efficiency (AUC = 0.86) during countermeasures.

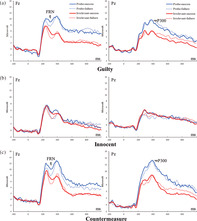

## 1 INTRODUCTION

The detection of deception and concealed information is fundamental to many fields, including national security and forensic science. The use of such techniques to identify perpetrators of atrocities may avert tragedy and save hundreds of lives. As such, the development of reliable and effective deception detection methods has attracted the attention of researchers from many different disciplines including psychology, law, and neuroscience (Rosenfeld, 2018).

With recent advances in neuroscientific methodology, researchers have begun to explore neurophysiological correlates of concealed information/deception by using event‐related potentials (ERPs) or functional magnetic resonance imaging (fMRI) to inform the development of concealed information tests (Gamer, 2011; Rosenfeld, 2020). In the field of ERP‐based CIT, there has been intensive research undertaken into an endogenous component, termed P300 (Rosenfeld, 2020). P300 is a positive ERP component that typically occurs 300–800 ms after the onset of a stimulus, and it is often elicited by infrequent, familiar, and significant stimuli (Johnson Jr, 1986; Polich, 2007). During a typical P300‐based CIT, an infrequent crime‐related stimulus (probe) was presented within a series of more frequent crime‐irrelevant stimuli. If the probe holds great meaning to a guilty suspect, it will elicit a larger P300 than is elicited by the other irrelevant stimuli. Conversely, an innocent suspect attributes no meaning to the probe and will perceive the probe to be just another irrelevant stimulus, and thus, there is no significant increase in the P300 amplitude between the probe and irrelevants. As such, the P300‐based CIT is used in the detection of crime‐related memories, not of deception itself.

The P300‐based CIT effectively detects concealed information, as demonstrated by a recent meta‐analysis (AUC = 0.88, Meijer, Selle, Elber, & Ben‐Shakhar, 2014). However, the P300‐based CIT relies solely on the detection of a memory or the recognition of a crime‐related stimulus by a perpetrator. In recent years, researchers have also tried to explore ways to further improve the utility of P300‐based CIT techniques. For instance, it may be possible to increase a participant's attention to a crime‐related stimulus to enhance the detection efficiency of P300 (e.g., Rosenfeld, Hu, & Pederson, 2012; and recently Olson, Rosenfeld, & Perrault, 2019). Furthermore, it may be possible to identify and utilize other ERPs that are independent of P300, such as N200 (which is thought to be associated with response monitoring processes) (Gamer & Berti, 2010; Hu, Pornpattananangkul, & Rosenfeld, 2013), or Medial Frontal Negativity, to increase the efficiency of the CIT (Scheuble & Beauducel, 2020).

Sai et al. (2016) introduced a novel ERP‐based CIT called the feedback‐CIT (fCIT). In the fCIT, participants are provided with feedback on their memory concealment performance following each trial. This paradigm not only retains the advantages of the recognition‐related P300 in detecting concealed information but can use feedback‐related ERPs to improve detection efficiency. The study focused on two components of feedback‐related ERPs; the feedback‐related negativity (FRN) and the feedback‐P300. In the early literature, negative feedback was typically associated with a more negative ERP component occurring around 200–350 ms following the feedback stimulus, whereas positive feedback evoked no such response (e.g., Gehring & Willoughby, 2002; Yeung & Sanfey, 2004). However, some recent findings reported the use of principal component analysis (PCA) to determine that the FRN reflects a reward‐related positivity that is absent or suppressed following monetary loss (for a review, see Proudfit, 2015).^1^ The feedback‐P300 which follows the FRN is a positive ERP component that occurs around 300–500 ms after a feedback stimulus. Although the feedback‐P300 is thought to be functionally separate from the FRN (Yeung & Sanfey, 2004), there is evidence that feedback elicited larger FRN and feedback‐P300 amplitudes when the participant had a motivation to deceive (e.g., Sai, Wu, Hu, & Fu, 2018). Furthermore, Sai et al. (2016) reported that feedback following a probe stimulus produced more positive FRN and feedback‐P300 amplitudes than feedback following an irrelevant stimulus in participants who intended to conceal. However, the FRN and feedback‐P300 elicited by feedback following the probe were not significantly different from those elicited by the feedback following irrelevant stimuli in participants who did not have the intention to conceal. Analyses on the individual level revealed that the FRN and feedback‐P300 are both effective in the identification of participants who were and were not intending to conceal (AUC ≥ 0.95).

As identified in the report of the National Research Council of the National Academy of Sciences, one of the weaknesses of currently existing lie detector methodology is that deception detection tests are vulnerable to the influence of mental countermeasures (National Research Council, 2003). Rosenfeld, Soskins, Bosh, and Ryan (2004) and other researchers reported that the 3‐stimulus P300‐based CIT is also vulnerable to countermeasures. To increase the countermeasure resistance of the P300‐based CIT, Rosenfeld et al. (2008) developed a new P300‐based CIT called the complex trial protocol (CTP). The CTP separates probe or irrelevant recognition from target or nontarget discrimination to remove the element of dual‐task competition and thus facilitates a larger probe P300 resulting in increased resistance to countermeasure use. The CTP has been demonstrated to resist various countermeasures (Lukács et al., 2016; Rosenfeld et al., 2008; Sokolovsky, Rothenberg, Labkovsky, Meixner, & Rosenfeld, 2011). As noted above, the advantage of the fCIT is to not only use the recognition P300, but also to use feedback‐related ERPs (e.g., FRN and feedback‐P300) to detect concealed information. Although previous studies have shown that the fCIT provides an excellent detection efficiency, it remains unclear whether the fCIT can still maintain this high efficiency during the use of countermeasures.

To address this question, participants were trained to use countermeasures during the fCIT. The countermeasures involved making irrelevant items meaningful. Previous research has shown that this countermeasure can significantly decrease the accuracy of a 3‐stimulus P300‐based CIT (Labkovsky & Rosenfeld, 2012; Meixner, Haynes, Winograd, Brown, & Rosenfeld, 2009). It remains unclear whether this same mental countermeasure can also be used to decrease the accuracy of the fCIT. We hypothesized that the countermeasure would decrease the detection efficiency of recognition‐P300 in the fCIT by increasing the salience of irrelevant stimuli and reducing the relative salience of the probe in comparison (Hu, Hegeman, Landry, & Rosenfeld, 2012; Labkovsky & Rosenfeld, 2012; Meixner et al., 2009). Conversely, we hypothesized that the countermeasure would not influence the detection efficiency of feedback‐related ERPs because both the FRN and feedback‐P300 are related to participants’ motivation to conceal, rather than stimulus salience.

## 2 METHODS

### 2.1 Participants

A power analysis was conducted to determine the required sample size using G*Power 3.1 with power (1‐β) set at 0.95 and α = 0.05 (Faul, Erdfelder, Buchner, & Lang, 2009). Consequently, 20 participants were required in each group to detect a stimulus × group interaction with a medium effect size (effect size *f* = 0.20). Considering an attrition rate due to excessive artifacts, we thus recruited 90 participants (*n* = 25 male), with mean (±standard deviation) age of 21.6 (±1.8) years. Participants were randomly assigned to three groups: (a) guilty group (*n* = 30, 7 male; age = 21.7 ± 1.8 years); (b) innocent group (*n* = 30, 9 male; age = 21.7 ± 1.9 years); and (c) guilty group with countermeasures (*n* = 30, 9 male, age = 21.4 ± 1.8 years). One participant in each group was excluded from the ERP analyses due to excessive electrooculogram (EOG) artifacts. All participants had normal or corrected‐to‐normal vision, reported no history of neurological or psychological disorders, and were right‐handed. The protocol was approved by the ethics committee of Hangzhou Normal University and participants gave written informed consent prior to participation.

### 2.2 Procedure

Participants allocated to the guilty and guilty + countermeasures groups were asked to enact a mock crime: to enter a laboratory and steal a valuable objective (a ring) from an envelope in a drawer. Participants allocated to the innocent group were asked to walk around the same room without committing any crime. A wallet was put on the desk so that participants from all three groups would notice it, and was used as a target during the subsequent fCIT.

There were six stimuli in the fCIT: wallet, ring, watch, necklace, bracelet, and earring. The ring was the probe stimulus, the wallet was the target, and the remaining were irrelevant stimuli.

Following familiarization with the crime scene, the participant was taken to the electroencephalography (EEG) suite for the fCIT. During the fCIT, each of the six stimuli was presented one by one to the participant. The participant was instructed to respond by pressing the “F” key if they recognized the item, and the “J” key if they did not recognize the item. As all participants were exposed to the wallet (target), all participants were instructed to respond by pressing the “F” key, meaning “I recognize this item”. As all participants were not exposed to the four irrelevant items, all participants were instructed to press the “J” key, meaning “No, I don't recognize this item.” Participants from both guilty and countermeasure groups were further instructed to try to conceal the knowledge of the probe, requiring them to press the “J” key in response to it. Participants in the countermeasure group were additionally instructed to make a secret additional response for two of the four irrelevant stimuli. These two countermeasure responses took the form of the participant silently imagining their first name and last name (also see Hu et al., 2012; Labkovsky & Rosenfeld, 2012).

Participants were told that the test would provide feedback following each trial. Specifically, there were two possible feedback outcomes: a “+4” indicated that participants were telling the truth, whereas a “−2” indicated that participants were telling a lie. Unknown to participants, the feedback following the probe and irrelevant stimuli was bogus and was given randomly, while the feedback following the target was based on their actual performance. This approach was taken because it was thought that giving accurate feedback on their response to the target would be sufficient to convince the participants that all feedback provided was based on their EEG, and not random. This may be very important for the detection efficiency of the fCIT. The purpose of choosing “+4” and “−2” as the format of the feedback was to attempt to equalize the value of the gain and loss, in the context of previously reported findings showing that people are more sensitive to losses than to gains (Bress & Hajcak, 2013; Tversky & Kahneman, 1992). Key‐presses were counterbalanced across the three groups.

**FIGURE 1:**
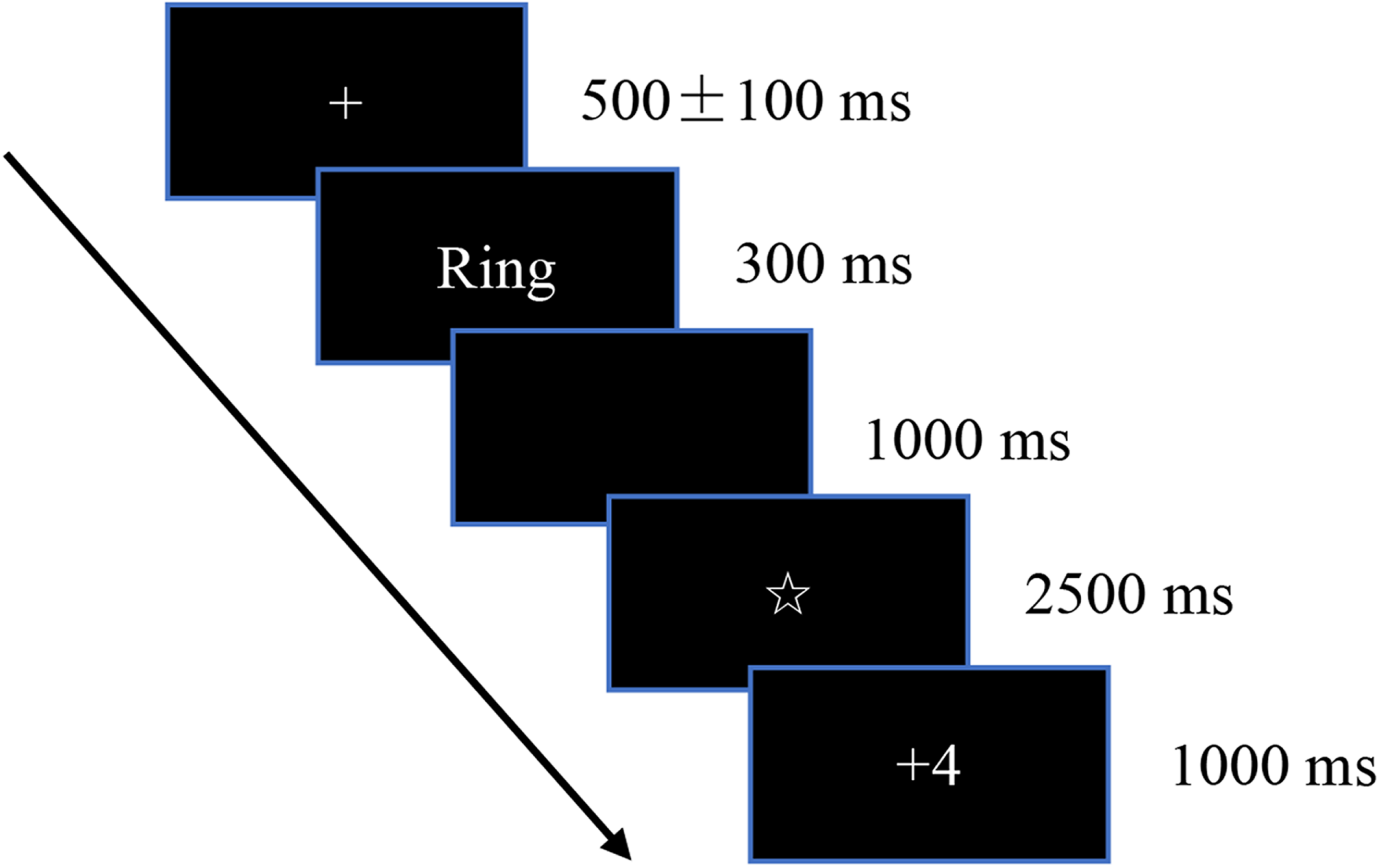
The task structure of the feedback concealed information test

Participants were seated approximately 1 m in front of the computer and were instructed to place their right index finger on the “J” key and left index finger on the “F” key. Each stimulus was presented in white font on a black background. Each trial began with a 500 ± 100 ms fixation point. Then a stimulus was presented in the middle of the screen for 300 ms, followed by a black screen for 1,000 ms. Participants were instructed to press one of two buttons as quickly and accurately as possible (see Figure 1). After the 1,000 ms blank screen, a star (“☆”) appeared for 2,500 ms, signifying that the lie detector was analyzing the participants’ real‐time EEG. Finally, the feedback was presented as “+4” or “−2” for 1,000 ms. As such, there were four feedback conditions: probe “+4” (success); probe “−2” (failure); irrelevant “+4” (success); irrelevant “−2” (failure). Each stimulus was repeated 60 times, with 50% of stimuli being followed by the feedback “+4”, and the other 50% followed by the feedback “−2”. There were 6 × 60 = 360 trials in total. Every 40 trials (approximately 2 min), the participant was allowed to take a break. The whole experiment lasted approximately 40 min.

### 2.3 EEG acquisition

EEG was recorded at 32 scalp sites using Ag/AgCl electrodes embedded in an elastic EEG cap (Brain Products, Germany). The cap was placed according to the 10–20 system, with the reference electrode on the right mastoid, and re‐referenced offline to the average signal of the left and right mastoid electrodes. Electrode impedances were kept below 10 KΩ. The sampling rate was 1,000 Hz.

BrainVision Analyzer (Brain Products, Germany) was used to conduct data analysis offline. The Ocular Correction independent component analysis (ICA) was used to remove artifacts associated with eye movement. Analysis of ERPs time‐locked to stimuli (i.e., probe vs. irrelevant), was focused on the P300 measured at the Pz electrode. Continuous EEG data in this stimuli stage were bandpass filtered at 0.16–6 Hz following the procedure of Rosenfeld (2020) and Rosenfeld, Hu, Labkovsky, Meixner, and Winograd (2013). Continuous EEG data were then segmented into epochs of 1,500 ms duration, including a 200 ms pre‐stimulus baseline, and time‐locked to the onset of the probe or irrelevant stimulus. This longer epoch length was chosen for the analysis of recognition‐P300 because the peak‐to‐peak (p–p) method subsequently used for calculating the shape of the P300 searches for the maximum negative 100‐ms segment after the P300 latency, which usually occurs over 1,000 ms after the stimulus (e.g., Olson et al., 2019; Winograd & Rosenfeld, 2014). Epochs were baseline‐corrected and trials with signals exceeding ±100 μV were defined as artifact trials and excluded. ERPs in response to irrelevant stimuli were averaged across all four irrelevant items. The numbers of trials in each condition of each group are presented in Table 1.

**TABLE 1:** The number of averaged trials in each condition of each group

	Guilty	Innocent	Countermeasure
Probe	51.72 (1.04)	51.07 (1.10)	48.79 (1.26)
Irrelevant	207.83 (4.34)	200.10 (4.32)	193.83 (5.20)
Probe‐success	25.34 (0.66)	24.97 (0.75)	24.79 (0.60)
Probe‐failure	25.86 (0.55)	25.14 (0.68)	24.14 (0.64)
Irrelevant‐success	104.03 (2.14)	100.41 (2.04)	98.76 (2.72)
Irrelevant‐failure	102.86 (2.34)	99.03 (2.12)	97.21 (2.61)

For ERPs time‐locked to the stimulus, the p–p method was used to identify and characterize the recognition‐P300. First, the p–p method searched for the maximally positive 100 ms segment from the range 300–800 ms, and the midpoint of this 100‐ms segment was defined as the P300 latency. Then the p–p method searched for the maximally negative 100 ms segment in the range between the P300 latency and 1,300 ms post‐stimulus. The “peak‐to‐peak” value is the amplitude difference between the average amplitudes of the maximally positive segment and the maximally negative segment (following the procedure outlined in Olson, Rosenfeld, Kim, & Perrault, 2018).

Analysis of ERPs time‐locked to the feedback was focused on the FRN and feedback‐P300. As these two ERP components largely overlap, a temporal PCA was conducted using ERP‐PCA toolkit version 2. 86 (Dien, 2010). In pre‐processing, continuous EEG data were bandpass filtered at 0.1–30 Hz on the basis that the FRN is composed of activity in the beta and theta bands, that range from 6 to 30 Hz, whereas the feedback‐P300 is composed of delta‐band activity in the frequency range 0–6 Hz (e.g., Li, Baker, Warren, & Li, 2016; Wang, Cheung, Yee, & Tse, 2020). Continuous EEG data were further segmented into epochs of 1,200 ms duration, containing a 200 ms pre‐stimulus baseline and a 1,000 ms time window after the onset of the feedback stimulus. Segments were baseline‐corrected, and trials with signals exceeding 100 μV were excluded. The PCA was conducted by combining data from the guilty, innocent, and countermeasure groups. The temporal PCA used the Promax Rotation method (Dien, Khoe, & Mangun, 2007), with the 1,200 time points of each participant's average ERP as variables (200 ms before the feedback starts as the baseline), and participants and conditions as observations. According to the scree plots, a total of 14 factors could be extracted, of which 6 factors account for more than 1% of the total variance of the data, which meets the standard. Given that the FRN is thought to occurs in the interval 200–350 ms, and the feedback‐P300 in the interval 400–600 ms, the positive component that peaked at 242 ms was selected as the FRN, and the positive component that peaked at 458 ms was selected as the feedback‐P300. The waveforms for each factor were reconstructed (i.e., converted to microvolts) by multiplying the factor pattern matrix by the matrix constructed from the standard deviations of each factor. Factors were then scored using the peak values (Foti, Weinberg, Dien, & Hajcak, 2011), which were applied to the subsequent analyses. The PCA factors selected for statistical analysis are listed in Table 2.

**TABLE 2:** The PCA factors selected for statistical analysis

Corresponding ERP component	Temporal factors	Temporal loading peaks (ms)	Variance explained (%)
FRN	TF03	242	5.15
Feedback P300	TF01	458	10.06

### 2.4 Statistical analysis

Analyses were performed using SPSS version 20.0. In the ANOVA, if the sphericity assumption was violated, the Greenhouse‐Geiser correction was used. For Post hoc comparisons, Fisher's least significant difference procedure was used. The effect size of the significant effect was expressed using partial eta squared and Cohen's *d*. In addition, Jeffreys–Zellner–Siow (JZS) Bayes factors (BFs) (scale *R* = 0.707; see Rouder, Speckman, Sun, Morey, & Iverson, 2009; http://pcl.issouri.Edu/bayesfactor) were reported to supplement classical statistical inference. The BF is a number value that serves as a method of quantifying the ratio of the likelihood of the null hypothesis relative to the likelihood of the alternative hypothesis. BFs are reported favoring either the null hypothesis or favoring the alternative hypothesis (Jeffreys, 1998; Kass & Raferty, Kass & Raftery, 1995). For all *t*‐tests, either the BF_10_ (favoring the alternative hypothesis) or the BF_01_ (favoring the null hypothesis) is reported. For all ANOVA effects, that is, both main and interaction effects, either the BF_Inclusion_ (favoring the alternative hypothesis) or BF_Exclusion_ (favoring the null hypothesis) is reported, reflecting a comparison of all models containing a particular effect to those without the effect (also see Klein Selle, Gueta, Harpaz, Deouell, & Ben‐Shakhar, 2021). A BF value of ≥3 was regarded as moderate evidence for the respective hypothesis (Kass & Raftery, 1995). BFs were computed using JASP (Version 0.14.1, https://jasp-stats.org/).

Lastly, receiver operating characteristic (ROC) analyses were conducted to examine the detection efficiency of each ERP component. ROC analyses, which are based on signal detection theory, describe detection efficiency by comparing the distributions of detection scores between guilty participants and innocent participants (Ben‐Shakhar & Elaad, 2003). An ROC curve is generated based on these distributions, and the area under the curve (AUC) is used to represent the detection efficiency of a test. The AUC varies between 0 and 1. An AUC of 0.5 means that the distribution of the detection score for guilty participants is not different from the distribution of the detection score for innocent participants, and an AUC of 1 means that the two distributions do not overlap at all, thereby providing a high detection efficiency (Meijer et al., 2014). ROC analyses in the present study were conducted based on the probe minus irrelevants P300, FRN, and feedback‐P300 in each group. In addition, to examine whether combing these indices would further improve efficiency, the probe mimus irrelevant values for each ERP component were transformed into stardard *z* scores across participants, and then *z* scores from each ERP component were averaged into a single measure (see also Hu et al., 2013).

## 3 RESULTS

### 3.1 Recognition‐P300s to probe vs. Irrelevants (see Figure 3)

**FIGURE 2:**
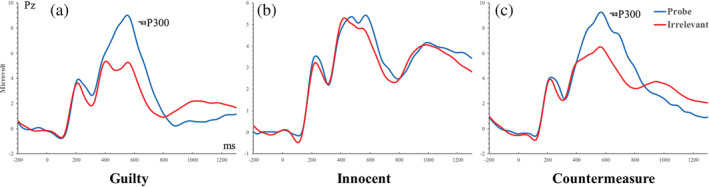
Grand‐average ERP waveforms evoked by the probe and irrelevant stimuli as measured at the Pz electrode

A two‐way mixed ANOVA using the three groups (between‐subject: guilty vs. innocent vs. countermeasure) and two stimulus types (within‐subject: probe vs. irrelevant) was conducted on the P300 amplitudes. Results showed a significant main effect of stimulus type, *F* (1, 84) = 34. 27, *p* < .001, *η*_*p*_^2^ = 0.29, BF_Inclusion_ = 2.20 × 10^6^, with the amplitude of the P300 elicited by the probe stimulus larger than that elicited by the irrelevant stimuli (7. 95 ± 0.65 μV vs. 4.95 ± 0.39 μV). There was a significant interaction between stimulus type and group, *F* (2, 84) = 10.91, *p* < .001 *η*_*p*_^2^ = 0.21, BF_Inclusion_ = 850.44. Post hoc tests showed that the probe stimulus produced a significantly greater P300 amplitude than did the irrelevant stimuli in the guilty group (10.65 ± 1. 12 μV vs. 4. 57 ± 0.67 μV, *t* (28) = 6.84, *p* < .001, *d* = 1.17, 95% CI = [3.40, 8.77], BF_10_ = 7.99 × 10^4^), and countermeasure group (8. 23 ± 1. 12 μV vs. 5. 53 ± 0.67 μV; *t* (28) = 3.04, *p* = .05, *d* = 0.50, 95% CI = [0.02, 5.39], BF_10_ = 8.10). There was no significant difference in P300 amplitude found between the probe and irrelevant stimuli in the innocent group (4. 98 ± 1. 12 μV vs. 4. 75 ± 0.67; *t* (28) = 0.26; *p* > .05, *d* = 0.05, 95% CI = [−2.46, 2.92], BF_01_ = 4.91).

To further examine the influence of mental countermeasures on the recognition‐P300, a two‐way ANOVA was conducted using the two groups (between‐subject: guilty vs. countermeasure) and two stimulus types (within‐subject: probe vs. irrelevant) on the P300 amplitudes. The results showed a significant interaction between stimulus type and group, *F* (1, 56) = 5.36, *p* = .02 *η*_*p*_^2^ = 0.09, BF_Inclusion_ = 2.41. Follow‐up independent‐sample *t*‐test showed that the difference between the probe and irrelevants (probe‐irrelevants) was smaller in the countermeasure group than that in the guilty group (2.71 ± 0.75 vs. 6.08 ± 1.25, *t* (56) = 2.32, *p* = 0.02, *d* = 0.61, 95% CI = [0.44, 6.32]). This result suggests that there is an effect of countermeasure on the recognition‐P300, but it was not strongly supported by the BF_10_ = 2.40. For grand averaged ERPs that are time‐locked to CIT stimuli, see Figure 2.

**FIGURE 3:**
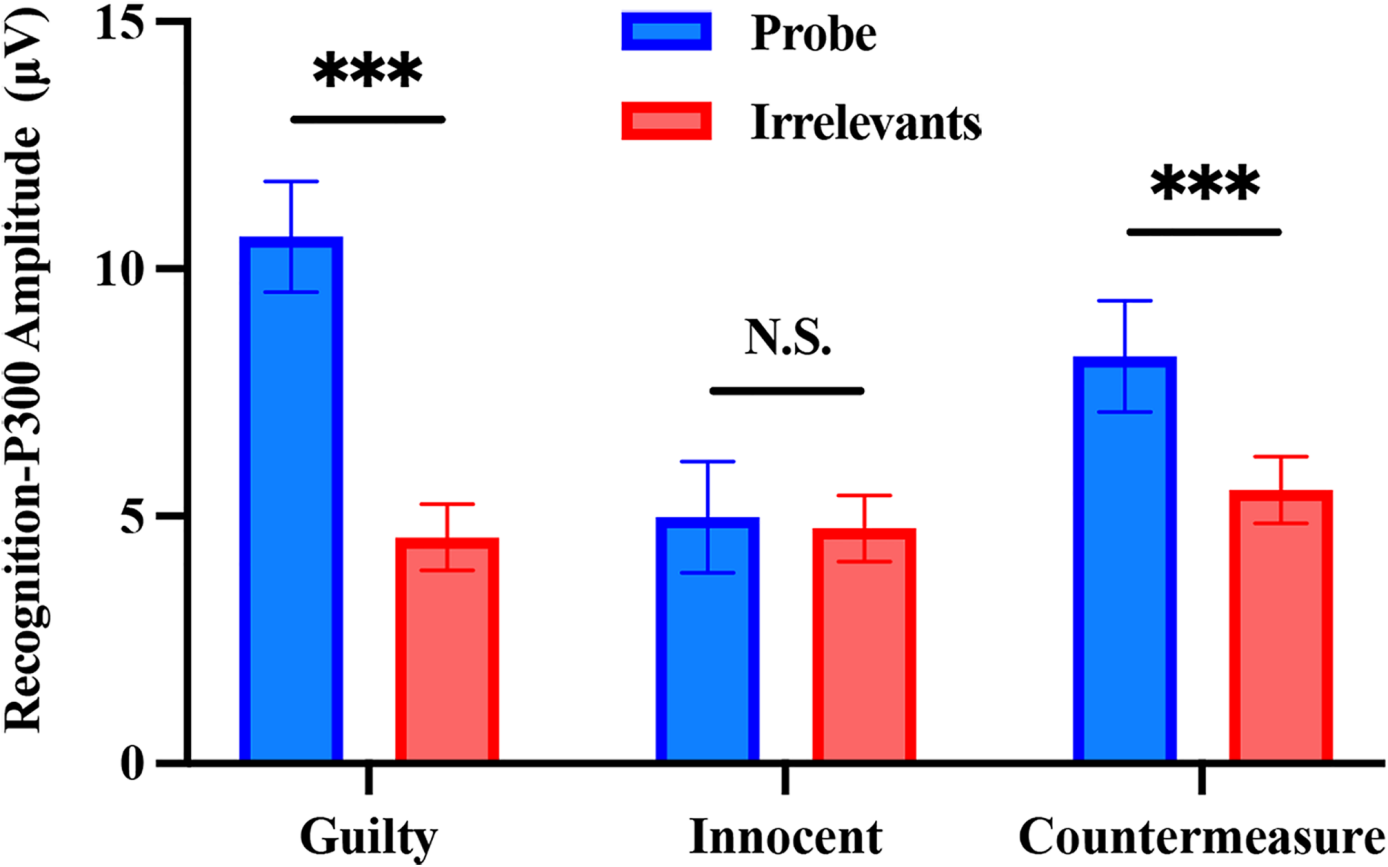
The mean peak‐to‐peak recognition‐P300 amplitude as a function of stimulus in each group (*** denotes significance at a level of *p* < .001)

### 3.2 FRN at Fz (a positivity peaking at 242 ms corresponded to FRN, see Figure 5)

**FIGURE 4:**
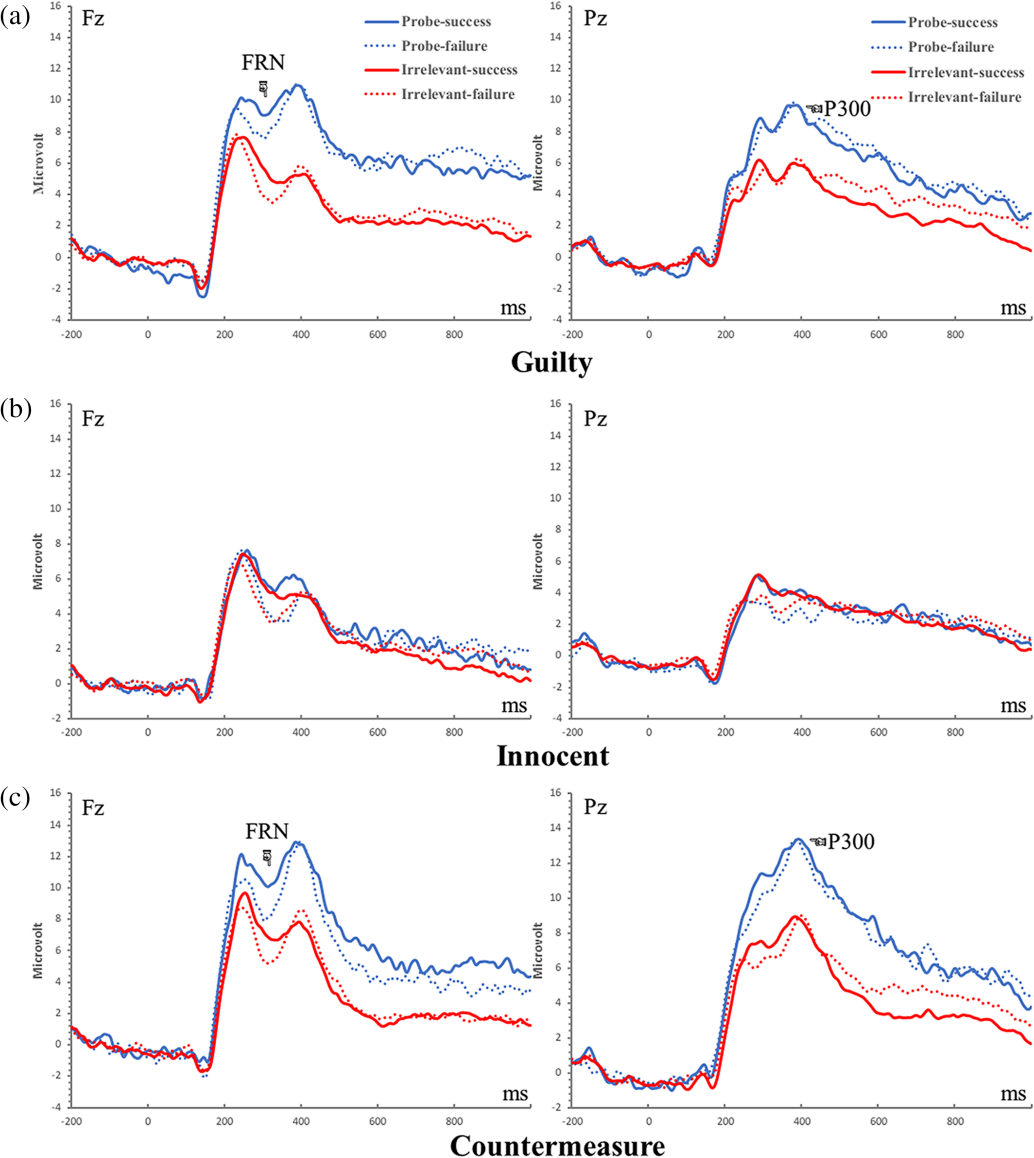
The grand‐average event‐related potentials waveforms from the Fz and Pz electrodes (before PCA transformation) during the feedback stage, by group

A three‐way mixed ANOVA was conducted on the FRN amplitude using two stimulus types (probe vs. irrelevant) by two feedback types (success vs. failure) by three groups (guilty vs. innocent vs. countermeasure). There was a significant main effect of stimulus type, *F* (1, 84) = 33. 91, *p* < .001, *η*_*p*_^2^ = 0.29, BF_Inclusion_ = 3.09 × 10^6^, with the probe stimulus eliciting a more positive FRN than irrelevant stimuli (5. 88 ± 0.56 μV vs. 4. 41 ± 0.42 μV). There was also a significant main effect of feedback type, *F* (1, 84) = 5. 62, *p* = .02, *η*_*p*_^2^ = 0.06, BF_Inclusion_ = 1.34, with successes eliciting a more positive FRN than failures (5. 46 ± 0.52 μV vs. 4. 84 ± 0.47 μV). But this effect was not supported by BF_Inclusion_ = 1.34. The interaction between stimulus type and group was also significant: *F* (2, 84) = 4. 86, *p* = .01, *η*_*p*_^2^ = 0.10, BF_Inclusion_ = 7.32. Post hoc tests indicated that in the guilty and countermeasure groups, the probe stimulus induced a greater FRN in comparison to the irrelevant stimuli (Guilty goup: 6. 14 ± 0.98 μV vs. 4. 10 ± 0.73 μV; *t* (28) = 4.65, *p* < .001, *d =* 0.45*,* 95% CI = [0.71, 3.35], BF_10_ = 350.83; Countermeasure group: 7. 50 ± 0.98 μV vs. 5. 48 ± 0.73 μV; *t* (28) = 4.62, *p* < .001, *d =* 0.34*,* 95% CI = [0.70, 3.34], BF_10_ = 325.79). Conversely, there was no difference in FRN amplitude between responses to the probe and irrelevant stimuli in the innocent group (4.01 ± 0.98 μV vs. 3. 65 ± 0.73 μV; *t* (28) = 0.82, *p* > .05, *d =* 0.12*,* 95% CI = [−0.96, 1.68], BF_01_ = 3.72. There were no other significant main effects or interactions (stimulus × feedback: *F* (1, 84) <1, *p* > .05, BF_Exclusion_ = 4.28; feedback × group: *F* (2, 84) < 1, *p* > .05, BF_Exclusion_ = 5.33; stimulus × feedback × group: *F* (2, 84) = 1.05, *p* > .05, BF_Exclusion_ = 30.20). For grand averaged ERPs and their scalp distributions and elicited by feedback stimuli, see Figures 4 and 6a).

To examine the effect of mental countermeasures on the FRN, a two‐way ANOVA was conducted using the two groups (between‐subject: guilty vs. countermeasure) and two stimulus types (within‐subject: probe vs. irrelevant) on FRN amplitude. The interaction between stimulus type and group was not found to be significant, *F* (1, 56) = 0.04, *p* > .05 *η*_*p*_^2^ < 0.01, which suggested no effect of countermeasure on the FRN. It should be noted that this result was not strongly supported by BF_Exclusion_ = 2.49.

### 3.3 Feedback‐P300 at Pz (corresponding to a positive ERP component peaking at 458 ms, see Figure 5)

**FIGURE 5:**
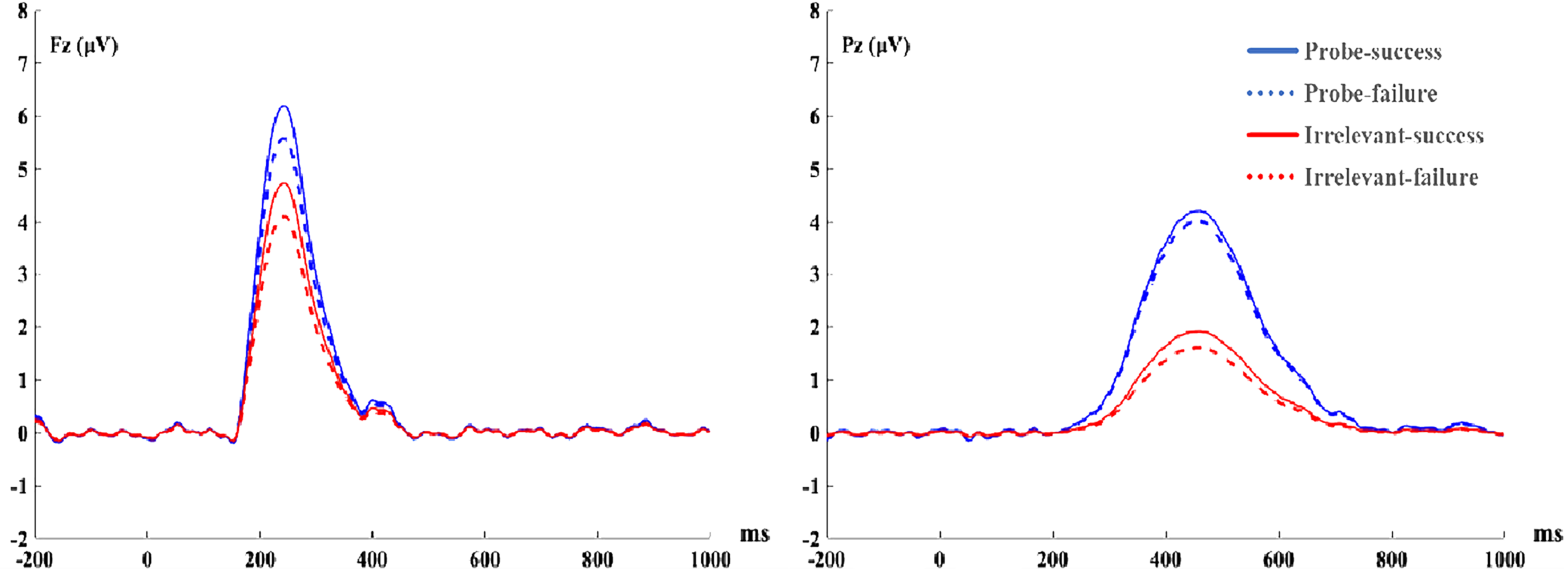
PCA‐extracted ERP waveforms during the feedback stage, combining guilty, innocent, and countermeasure groups

**FIGURE 6:**
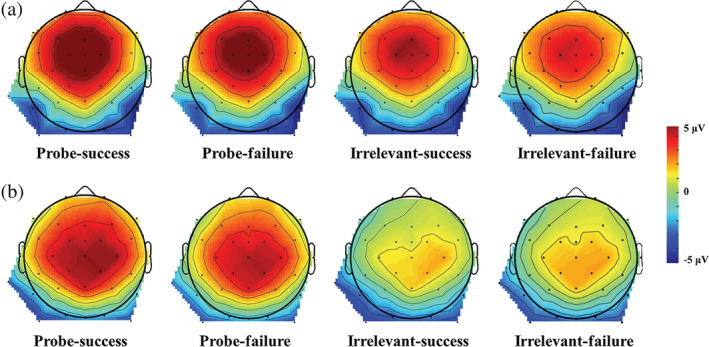
PCA‐based scalp distributions of (a) the FRN and (b) the feedback‐P300 during the feedback stage, combining guilty, innocent, and countermeasure groups

A three‐way mixed ANOVA was performed on the amplitude of feedback‐P300 with the two stimulus types (probe vs. irrelevant) by two feedback types (successes vs. failures) by three groups (guilty vs. innocent vs. countermeasure). There was a significant main effect of stimulus type, *F* (1, 84) = 46.10, *p* < .001, *η*_*p*_^2^ = 0.35, BF_Inclusion_ = 5.36 × 10^14^, with a larger feedback‐P300 following feedback on their response to the probe stimulus than to the irrelevant stimuli (4. 11 ± 0.60 μV vs. 1. 77 ± 0.48 μV). There was also a significant interaction between stimulus type and group, *F* (2, 84) = 14. 72, *p* < .001, *η*_*p*_^2^ = 0.26, BF_Inclusion_ = 5.93 × 10^8^. Post hoc tests revealed that feedback‐P300 associated with the probe stimulus was larger than that associated with irrelevant stimuli in both the guilty and countermeasure groups (guilty group: 4. 89 ± 1.03 μV vs. 1. 70 ± 0.83 μV, *t* (28) = 5.34, *p* < .001, *d =* 0.54*,* 95% CI = [1.38, 4.99], BF_10_ = 1.95× 10^3^, and countermeasure group: 7. 49 ± 1.03 μV vs. 3. 40 ± 0.83 μV, *t* (28) = 6.84, *p* < .001, *d =* 0.76*,* 95% CI = [2.28, 5.89], BF_10_ = 7.99 × 10^4^), but not in the innocent group (−0.04 ± 1.03 μV vs. 0.21 ± 0.83 μV, *t* (28) = −0.42, *p* > .05, *d =* 0.07*,* 95% CI = [−2.06, 1.55], BF_01_ = 4.67). Other interactions were nonsignificant: stimulus × feedback: *F* (1, 84) = 2. 18, *p* > .05, BF_Exclusion_ = 14.57; feedback × group: *F* (2, 84) = 1. 93, *p* > .05, BF_Exclusion_ = 16.92, stimulus × feedback × group: *F* (2, 84) = 0.40, *p* > .05, BF_Exclusion_ = 83.79. For grand averaged ERPs and their scalp distributions and elicited by feedback stimuli, see Figures 4 and 6b).

To examine the effect of mental countermeasures on the feedback‐P300, two‐way ANOVA was conducted using the two groups (between‐subject: guilty vs. countermeasure) and two stimulus types (within‐subject: probe vs. irrelevant) on the feedback‐P300 amplitude. The interaction between stimulus type and group was not found to be significant, *F* (1, 56) = 0.83, *p* > .05, *η*_*p*_^2^ = 0.02, which suggested no effect of countermeasure on the feedback‐P300. But it should be noted that this result was not supported by BF_Exclusion_ = 1.57.

### 3.4 Receiver operating characteristics

To examine how well the ERP components can be used to distinguish between participants in the guilty group from those in the innocent group, and from those using mental countermeasures, Receiver Operating Characteristics (ROC) analyses were conducted with the guilty, countermeasure, or innocent group as the state variable, and the difference values between the ERP components to the probe and irrelevant stimuli as the independent variable. As shown in Table 3, the recognition‐P300 can be effectively used to discriminate between participants in the guilty and innocent groups (AUC = 0.82, *p* < .001), but the detection efficiency decreased when participants in the guilty group used mental countermeasures (AUC = 0.69, *p <*. 05*)*. Further, both the FRN and feedback‐P300 can also be used to discriminate participants in the guilty group from those in the innocent group, and the use of countermeasures did not affect their detection efficiency. The ROC analyses based on all the combined measurement achieved the highest classification efficiency (AUC = 0.87, *p* < .001), but the classification efficiency decreased when using countermeasures (AUC = 0.79, *p* < .001). Notably, the ROC analyses demonstrated the feedback‐P300 alone to provide the highest classification efficiency when using countermeasures (AUC = 0.86, *p* < .001).

**TABLE 3:** AUCs of ERP components at distinguishing between groups

Group	ERP	AUC	95% CI
Guilty‐innocent	Recognition‐P300	0.82***	0.70–0.93
	FRN	0.70**	0.57–0.84
	Feedback P300	0.80***	0.68–0.91
	Recognition‐P300 + FRN	0.84***	0.74–0.94
	Recognition‐P300 + FP300	0.87***	0.78–0.96
	FRN + FP300	0.82***	0.71–0.93
	All indices	0.87***	0.78–0.97
Countermeasure‐innocent	Recognition‐P300 FRN	0.69* 0.69*	0.55–0.83 0.56–0.83
	Feedback P300	0.86***	0.76–0.95
	Recognition‐P300 + FRN	0.72***	0.59–0.86
	Recognition‐P300 + FP300	0.82***	0.71–0.93
	FRN + FP300	0.81***	0.69–0.92
	All indices	0.79***	0.67–0.91

Abbreviations: AUC, area under curve; CI, confidence interval.

* *p* < .05,

** *p* < .01,

*** *p* < .001.

## 4 DISCUSSION

This article investigates the effect of mental countermeasures on a novel form of the CIT incorporating the presentation of feedback, termed fCIT. Previous research has shown that the fCIT has a good detection efficiency in detecting concealed information (AUC = 0.85–0.99). The present study examined the retained efficiency of the fCIT when mental countermeasures were used.

The detection efficiency of the recognition‐P300 decreased (AUC = from 0.82 to 0.69) when guilty participants were asked to use mental countermeasures (i.e., recalling their own name). This countermeasure strategy makes two of the irrelevant stimuli salient to the participants, decreasing the difference in recognition‐P300 between probe and irrelevant stimuli and ultimately resulting in decreased detection efficiency. As expected, our results confirmed that the probe‐irrelevant difference in recognition P300 was significantly smaller for the countermeasure group compared to the guilty group. This finding is consistent with previous studies showing that the 3‐stimulus P300‐based CIT is vulnerable to this mental strategy (e.g., Rosenfeld & Labkovsky, 2010).

An advantage of the fCIT is that it can also incorporate information from the ERPs associated with the participant receiving feedback on their response to a stimulus to detect concealment of information. In agreement with previous research, both the FRN and the feedback‐P300 can be used to significantly distinguish participants concealing information (the guilty group in the present study) from those who were not (Sai et al., 2016, 2020; Sai, Lin, Hu, & Fu, 2014). Importantly, the use of a mental countermeasure strategy did not have a significant effect on the detection efficiency of either measure. In contrast, the detection efficiency of the feedback‐P300 slightly improved. Arguably, although feedback‐related ERPs are not sensitive to salient stimuli, participants in the countermeasure group may have paid more attention to the feedback following the two irrelevant stimuli that they applied the countermeasure to. For instance, they may have been interested to know whether the countermeasure they used was effective, thus increasing the FRN and feedback‐P300 following irrelevant stimuli. However, this argument was not supported by the results, considering that the interaction between the probe‐irrelevant P300 difference between the guilty and the countermeasure group was insignificant. Alternatively, participants in the two guilty groups may have cared more about the feedback they received following the probe stimulus regardless whether they used the countermeasures or not, given that they were instructed to conceal their familiarity with the probe stimulus and remain undetected by the experimenter.

Although feedback‐related ERPs during the feedback stage of the fCIT were resistant to the countermeasures used in the present study, this was not the case for the recognition‐P300 during the recognition stage. It should be noted that the CTP is a variant of the P300‐based CIT that has also been demonstrated to be resistant to mental countermeasures (see Rosenfeld et al., 2008; Rosenfeld & Labkovsky, 2010). Thus, one promising solution to this issue is to combine the complex trial protocol (CTP) with feedback. In this way, the detection efficiency of the recognition‐P300 may not be impaired by the use of countermeasures, but may also be improved by using both the recognition P300 and feedback‐related ERPs in combination to detect the concealment of information. Further investigation would be needed to explore the feasibility of this approach.

There are several limitations in the present study. First, the scope of the study was only to examine whether fCIT retains its efficacy for the detection of concealed crime‐related information when mental countermeasures are used. Further studies are needed to determine whether the fCIT is resistant to mental countermeasures when used to detect concealed autobiographical information such as names or dates of birth (e.g., Sai et al., 2014). Second, the present study only examined the effect of one mental countermeasure strategy on the fCIT. It is also necessary to examine whether the fCIT is resistant to other countermeasures, such as performing covert act in response to irrelevants (Rosenfeld et al., 2004, 2008) Third, most of the participants in the present study were female. Future investigations should balance the gender distribution of participants and explore whether there is a gender effect on detecting the concealment of information. Fourth, this was the first study to examine the influence of mental countermeasures on the fCIT, representing the early exploration of a new field. More studies are needed to confirm the validity of these findings. Lastly, In real‐life situations, crimes are often committed under great stress and time constraints, whereby the perpetrator may not perceive or encode information related to the crime effectively (Ben‐Shakhar & Nahari, 2018; Meijer, Verschuere, Gamer, Merckelbach, & Ben‐Shakhar, 2016). Thus, further studies should test the efficiency of the fCIT in field scenarios.

In conclusion, the present study demonstrated that the use of mental countermeasures impairs the detection efficiency of the recognition‐P300, but has no significant impact on feedback‐related ERPs. The feedback P300 continued to discriminate guilty and innocent participants with AUCs well above chance. These findings indicate the potential of the fCIT to subvert mental countermeasure strategies used in the concealment of important information.

## Supporting information


Figure S1‐S2

## Data Availability

The data that support the findings of this study are available on request from the corresponding author.

## ENDNOTE

^1^
Although some research suggests to call FRN as the reward‐related positivity (RewP), we keep using this old term to be consistent with our previous research.
