# Supplementary material for: The effect of mental countermeasures on a novel brain‐based feedback concealed information test
Source: Hum Brain Mapp. 2022 Feb 23;43(9):2771–81. doi: 10.1002/hbm.25814 (PMC9120554; doi:10.1002/hbm.25814)
Supplement: Supplementary file 1 — Figure S1‐S2 [file HBM-43-2771-s001.docx]

*Reaction time (see Figure S1)*

A two-way mixed ANOVA using the 3 groups (between-subject: guilty vs. innocent vs. countermeasure) and 2 stimulus types (within-subject: probe vs. irrelevant) was conducted on the reaction time. Results showed a significant main effect of stimulus types, *F* (1, 80) = 49. 81, *p* < .001, *η_p_^2^* = 0.38, BF_Inclusion_ = 3.05×10^8^, with the RT of probe stimulus longer than that of the irrelevant stimuli (522.55 ± 8.75 vs. 494.28 ± 9.27). There was a significant interaction between stimulus type and group, *F* (2, 80) = 15.72, *p* < .001 *η_p_^2^* = 0.28, BF_Inclusion_ =1.43×10^4^, Post hoc tests showed that the probe stimulus produced a longer RT than did the irrelevant stimuli in the guilty group (550.20 ± 15. 62 vs. 504.91 ± 16.55, *t* (25) = 4.80, *p* < .001, *d* = 0.42, 95% CI = [25.84, 64.74], BF_10_ = 407.16), and countermeasure group (526.29 ± 15. 05 vs. 483.68 ± 15.94; *t* (27) = 6.96, *p* < .001, *d* = 0.74, 95% CI = [30.06, 55.17], BF_10_ = 8.88×10^4^). There was no significant difference in RT found between the probe and irrelevant stimuli in the innocent group (491. 17 ± 14. 79 vs. 494. 27 ± 15.67; *t* (28) = -0.63; *p* > .05, *d* = -0.04, 95% CI = [-13.27, 7.07], BF_01_ = 4.22).


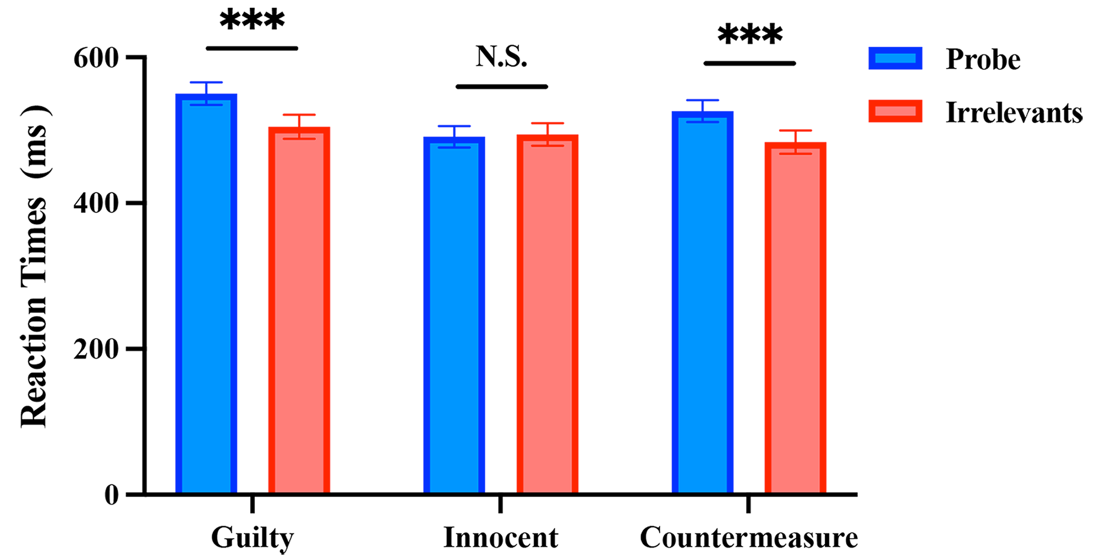


Figure S1. Mean ± SE of reaction times (RTs) from the probe and irrelevants in the fCIT.

*Accuracy (see Figure S2)*

A two-way mixed ANOVA using the 3 groups (between-subject: guilty vs. innocent vs. countermeasure) and 2 stimulus types (within-subject: probe vs. irrelevant) was conducted on the accuracy. Results showed a significant main effect of stimulus type, *F* (1, 80) = 20. 31, *p* < .001, *η_p_^2^* = 0.20, BF_Inclusion_ = 8.15×10^5^, with the ACC of probe stimulus lower than that of the irrelevant stimuli (0.989 ± 0.002 vs. 0.997 ± 0.001). There was a significant interaction between stimulus type and group, *F* (2, 80) = 5.46, *p* < .01 *η_p_^2^* = 0.12, BF_Inclusion_ = 101.85, Post hoc tests showed that the probe stimulus produced a lower ACC than did the irrelevant stimuli in the guilty group (0.981 ± 0.004 vs. 0.997 ± 0.001 , *t* (25) = -3.72, *p* < .001, *d* = -1.03, 95% CI = [-0.03, -0.01], BF_10_ = 33.99), and countermeasure group (0.990 ± 0.003 vs. 0.998 ± 0.001 ; *t* (27) = -2.64, *p* < .05, *d* = -0.72, 95% CI = [-0.01, -0.001], BF_10_ = 3.56). There was no significant difference in ACC found between the probe and irrelevant stimuli in the innocent group (0.996 ± 0.002 vs. 0.997 ± 0.001; *t* (28) = -0.49; *p* > .05, *d* = -0.13, 95% CI = [-0.01, 0.003], BF_01_ = 4.54).


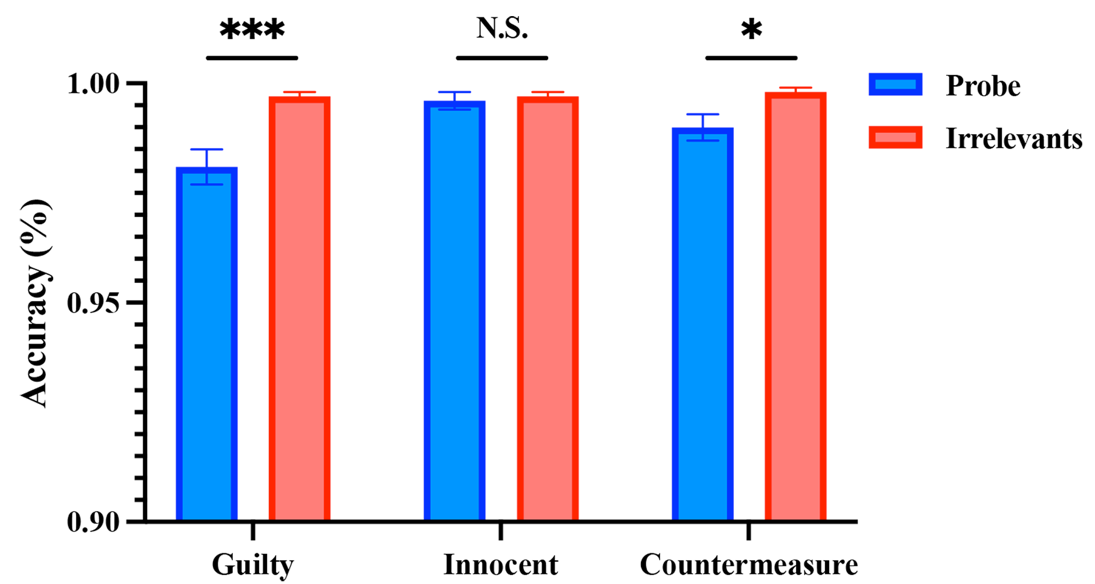


Figure S2. Mean ± SE of accuracy (%) from the probe and irrelevants in the fCIT.
